# A Narrative Review on Effects of Maternal Bariatric Surgery on Offspring

**DOI:** 10.7759/cureus.48513

**Published:** 2023-11-08

**Authors:** Mrunmayee M Pathak, Kartikey V Shekhar, Revat J Meshram

**Affiliations:** 1 Surgery, Jawaharlal Nehru Medical College, Datta Meghe Institute of Higher Education and Research (Deemed to Be University), Wardha, IND; 2 Paediatrics, Jawaharlal Nehru Medical College, Datta Meghe Institute of Higher Education and Research (Deemed to Be University), Wardha, IND

**Keywords:** gestational diabetes mellitus, obesity, offspring, nutrition, bariatric surgery

## Abstract

Bariatric surgery (BS) has emerged as an efficient approach for addressing obesity, offering long-term benefits encompassing substantial weight loss and improving metabolic disorders. Many women of childbearing age opt for BS to enhance their health and well-being. The weight loss achieved through these procedures can positively impact pregnancy outcomes, but it's crucial to consider potential drawbacks. Micronutrient deficiencies, such as anemia resulting from iron or vitamin B12 deficiency, are a legitimate concern. Making the decision to have a BS is a complex process with many possible obstacles. The complicated nature of this decision is highlighted by worries about dumping syndrome, surgical complications that could include the risk of internal hernias, and the possibility that infants could be labeled as small for gestational age because of maternal undernourishment. Furthermore, there is a notable absence of international consensus regarding the ideal timing for conceiving after undergoing BS. Therefore, this narrative review extensively explores the existing body of literature, offering insights into the prevailing challenges encountered before and during pregnancy following BS. These challenges encompass a wide range of considerations, commencing with fertility-related issues. The study will cover strategies for addressing vitamin and nutritional deficiencies through supplementation, subtleties of post-BS altered glucose metabolism and how it affects the detection and treatment of gestational diabetes, how dumping syndrome progresses, various surgical problems, and how different bariatric procedures affect pregnancy and fetal outcomes. These include a tendency to give birth to children considered undersized for gestational age, nutritional deficits, anemia, and abnormal maternal glucose metabolism. This review offers a comprehensive exploration of the multifaceted landscape of pregnancy in the context of BS. It aims to provide a valuable resource for healthcare professionals and women considering pregnancy after undergoing BS, enabling them to make well-informed decisions and receive appropriate care during this critical phase of life.

## Introduction and background

Sleeve gastrectomy (SG), Roux-en-Y gastric bypass (RYGB), and laparoscopic adjustable gastric banding (LAGB) treatments are all examples of bariatric surgery (BS). This medical technique changes the digestive system to reduce food intake and regulate calorie absorption. The sustained long-term weight loss advantages of RYGB make it unique [1]. However, SG is gaining popularity, particularly in cases where future family planning is a consideration [2]. Determining the most suitable surgical procedure for optimizing pregnancy-related outcomes remains a subject of ongoing investigation, as there is no conclusive evidence to guide this decision [1,3].

The surge in bariatric surgeries over recent decades, coupled with the well-established adverse effects of obesity on pregnancy, has prompted growing interest in pre-pregnancy bariatric procedures and their potential influence on pregnancy outcomes [2]. A decrease in the incidence of gestational diabetes, hypertensive disorders, preeclampsia, and the delivery of large for gestational age (LGA) newborns has been shown in several research that has evaluated the effect of weight loss surgery in pregnancies [1,2]. Nevertheless, BS can lead to nutritional deficiencies, including folate, iron, and vitamin D, potentially affecting mothers and their infants [4]. These investigations often contrast pregnant women who had BS before pregnancy to those who did not but satisfied the requirements for such treatment [3].

Few studies have explored the long-term effects of maternal weight loss surgery, like dumping syndrome, nausea, dizziness, low blood sugar, and malnutrition, even though research has been done on how it affects obstetrical and short-term baby outcomes. A few small-scale sibling-analysis studies that compared siblings born before and after one ‘s mother's bariatric procedure have found a lower prevalence of childhood obesity in babies born just after the procedure [4]. Several of these trials have shown significant metabolic changes, such as better lipid profiles, relatively low levels of c-reactive protein, and higher levels of ghrelin. It is important to remember that not all research has shown a measurable impact on long-term weight development, and some data even point to a higher risk of pediatric hormonal illness following maternal weight loss surgery [5].

Small sample sizes are a common problem for research in this field, restricting their generalization. Additionally, the majority of research solely addresses endocrine morbidities, mainly obesity, and does not offer a thorough picture of the overall health consequences of the progeny. This review aimed to examine maternal BS's long-term impact on children's health. In contrast to other research, we only used a large-scale sibling analysis technique in our investigation [6]. By directly comparing siblings who were born before and after the mother's BS, this study's methodology was able to account for shared genetic and environmental variables [7].

## Review

Methodology

Search Strategies

A literature search in English was conducted using PubMed, Medline, Embase, Google Scholar, ResearchGate, and other electronic libraries of articles to examine the English-language literature.

Inclusion Criteria

Peer-reviewed journals produced in English, articles published in the last 50 years, the full text of the publication, type of publication: review articles, systematic review, meta-analysis, or empirical studies published in peer-reviewed scientific journals, compliance with the combinations of keywords: maternal BS, gestational diabetes mellitus, obesity, offspring, clinical features, pathobiology, treatment advances, targeted therapy, and the neurological manifestation.

Exclusion Criteria

Articles published earlier than 50 years, lack of full text of the publication, language of the publication different than English, type of publication other than review articles, systematic review, meta-analysis, or empirical studies published in peer-reviewed scientific journals, malignancy, co-morbidities along with the diseases affecting presentation, specific treatment protocol or than that of the illness in review.

**Figure 1:**
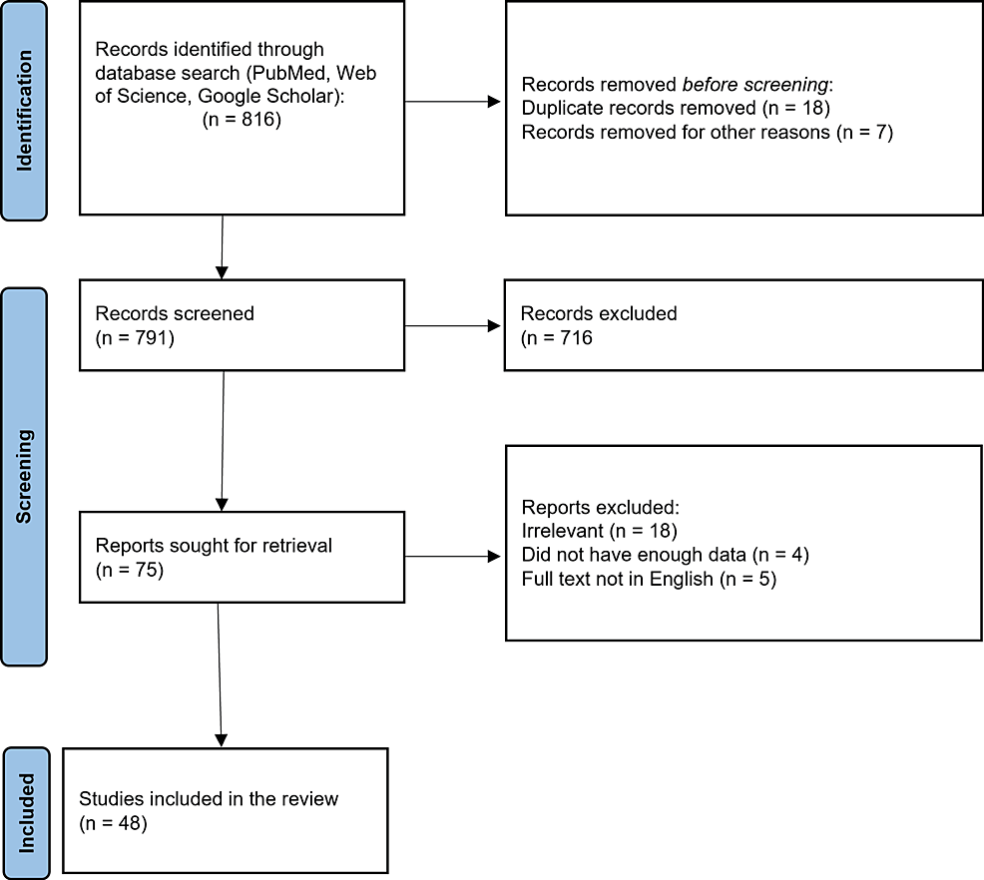
The selection process of articles used in this study Preferred reporting items for systematic reviews and meta-analysis (PRISMA) flowchart for the keywords used in the literature review

Key terms used for the search are "gestational diabetes mellitus" (all fields) or "bariatric surgery" (all fields) and "effects on offsprings" (all fields) or "gestational diabetes mellitus" (mesh terms) or "Roux-en-Y gastric bypass" (all fields). The preferred reporting items for systematic reviews and meta-analysis methods used in research methodology are depicted in Figure 1.

Overview of bariatric procedures

If traditional weight-loss methods have proven ineffective, BS may be necessary. Historically, it has demonstrated notable effectiveness in weight reduction, as well as in the mitigation of associated comorbidities such as type 2 diabetes and hypertension, while also enhancing heart function. International recommendations suggest considering BS for individuals with a BMI exceeding 40 kg/m^2^ [8]. Bariatric surgeries can be categorized into two primary types: restrictive and malabsorptive procedures or a combination [9]. Notably, the biliopancreatic diversion, a less common surgical approach, will not be discussed in this review. RYGB is a bariatric procedure that combines aspects of both malabsorptive and restrictive techniques. It involves horizontally dividing the upper part of the stomach to create a smaller gastric pouch. A biliopancreatic limb, typically ranging from 30 to 60 cm in length, is utilized to construct the alimentary limb, which allows ingested food to bypass a portion of the small intestine without the need for full biliopancreatic secretions, which are directed straight into the bowel via this limb [10]. For individuals seeking a restrictive approach, laparoscopic gastric resection, or sleeve gastrectomy, creates a smaller gastric pouch. In some cases, it may be combined with a duodenal ileostomy to form a biliopancreatic bypass. Additionally, an adjustable gastric band is placed approximately 1 to 2 cm below the gastroesophageal junction during a laparoscopic procedure. This band can be adjusted by injecting saline through a port, allowing for modulation of the level of stomach restriction [11]. This essay will overview bariatric surgeries, excluding biliopancreatic diversion and other less common procedures.

Minimally invasive endoscopic methods

Endoscopic placement of intragastric balloons, each having a minimum capacity of 400 ml, alters gastric motility and occupies space within the stomach. Bariatric endoscopy (BE) is less intrusive, more cost-effective, and linked to reduced morbidity and mortality than routine BS. It could also be permitted for individuals with a BMI of 30 to 35 kg/m^2^, depending on their circumstances. Furthermore, should more time be needed, the process can be reiterated. BE provides metabolic advantages, decreasing hyperuricemia, hypertriglyceridemia, hypercholesterolemia, and diabetes mellitus [12]. Thus far, there has been just a single retrospective study delving into the possible benefits of this method for individuals contending with infertility linked to obesity. Within this study, it was discovered that among 27 obese women who underwent intragastric balloon placement and subsequently achieved weight reduction, 15 successfully achieved conception. While these pregnancies proceeded without complications and resulted in healthy babies, it is crucial to emphasize that further research is necessary before we can confidently assert its safety during pregnancy and in women of reproductive age [13].

Obstacles and advantages of BS before conception

Although the frequency of BS in women of reproductive age has increased in recent years, and obesity has emerged as a significant healthcare issue, there is no global agreement on how to treat pregnancy after BS [14]. Rapid weight loss is expected the first year after surgery, and being pregnant during this catabolic period might affect the nourishment the developing fetus receives. Contrary to these recommendations, a recent investigation showed no proof in favor of this advice [15].

Anemia, poor protein and vitamin levels, and malabsorptive treatments are prevalent in post-BS pregnancies. Additionally, a history of BS affects the diagnosis of hyperglycemia due to impaired glucose metabolism. Research indicated a statistically insignificant trend toward more excellent stillbirth or newborn mortality rates. Children with small for gestational age (SGA) are at an increased risk, according to emerging information. Additionally, women who've had gastric bypass surgery before becoming pregnant are more prone to develop an internal hernia, which could have serious consequences, including intestinal necrosis or an immediate rupture, and necessitate an emergent cesarean birth [16]. There have also been some unusual occurrences of maternal and fetal mortality reported. The paragraphs that follow will go into further detail and address these topics.

Nutritional dimensions

Deficiency Anemia

Hemoglobin (Hb) and hematocrit levels typically drop during pregnancy, increasing blood volume by around 50% but an increase in red blood cell mass of only about 25%. During pregnancy, an extra 1,200 mg of iron is needed to fulfill the needs of the developing fetoplacental unit. Although the amount of iron absorbed increases during pregnancy, a healthy diet alone cannot satisfy all the demands, particularly for women with poor iron status before becoming pregnant (ferritin level 30 g/l) [17]. When Hb levels in pregnant women are less than 11 mg/dl, anemia is present, and iron deficiency is responsible for nearly 50% of cases. About 41.8% of this population group worldwide suffers from anemia. Neurobehavioral issues and an elevated risk of heart attack are among the long-term health effects of parental iron deficiency on children. The American College of Obstetricians and Gynecologists (ACOG) advises pregnant women without a history of BS to consume 30 to 60 mg of elemental iron daily and 27 mg of ferrous iron daily [18].

Hence, it is important to ensure that any prescribed dosage falls within this established safe range. Regular laboratory assessments are essential, and the results should guide any necessary dosage adjustments. At every phase of treatment, the ACOG recommends comprehensive blood tests encompassing iron and ferritin measurements [19,20]. During pregnancy, the daily demand for folic acid rises from 50 to 400 g, and diet could always provide this need. Following all BS treatments, folic acid insufficiency appears to be uncommon. Women with Crohn's disease or after gastric resection are likelier to get vitamin B12 deficient anemia. A 0.2 g/day increase in vitamin B12 intake is thought to be necessary during pregnancy. Because of reduced intrinsic factor and stomach acid production and bypassing of the duodenum, the primary site of absorption, vitamin B12 insufficiency appears to be more common following malabsorptive or combination BS. About 4-62% of people experience vitamin B12 shortage following RYGB, and the incidence tends to rise with time. This might be because the body's reserves can make up for the initial decline in absorption [21].

Metabolic processes related to glucose and gestational diabetes

Gestational diabetes mellitus (GDM) poses a significant health concern, affecting approximately 6% of European pregnancies. It is characterized by the onset of diabetes in the second or third trimester of pregnancy, which does not fit the criteria for preexisting type 1 or type 2 diabetes. To identify GDM, the most recent recommendations suggest using a two-hour, 75g oral glucose tolerance test (OGTT) during weeks 24+0 to 28+6 of gestation [22]. However, variations in diagnostic techniques and criteria have led to discrepancies in research findings and epidemiological data, rendering GDM diagnosis a subject of ongoing debate [22].

Obesity as a Risk Factor

Obesity emerges as a prominent risk factor for the development of GDM. Studies indicate that there may be a relationship between obesity and the risk of GDM. Women who are considered overweight in this situation and have a pre-pregnancy BMI between 25 and 30 have an odds ratio of 1.97. In a similar vein, women who are moderately obese with a BMI between 30 and 35 have an odds ratio of 3.01, and women who are severely obese with a BMI greater than 35 have a noticeably higher odds ratio of 5.55 in comparison to average weight women [23]. Sifting through the complex connections between obesity and GDM has been the focus of recent research. According to recent research, adipose tissue's release of pro-inflammatory cytokines may be a major factor in initiating systemic immunological and inflammatory disturbances, subsequently exacerbating maternal insulin resistance [23].

Adverse Pregnancy Outcomes and Metabolic Imprinting

GDM has been linked to adverse pregnancy outcomes, including macrosomia (considerable birth weight), preeclampsia, an increased likelihood of cesarean delivery, and large infants for their gestational age. The concept of "metabolic imprinting" is particularly concerning, where in-utero alterations in fetal organ function are attributed to excessive nutrient supply and heightened exposure to growth factors. This phenomenon appears to boost the risk of obesity and metabolic dysfunction in children born to diabetic mothers [23].

Impact of pre-pregnancy BS: Pre-pregnancy BS such as RYGB has demonstrated a significant potential to lower the risk of developing GDM. Women who underwent BS had roughly half the incidence of GDM compared to controls. However, it's important to note that outcomes vary depending on diagnostic criteria and control groups used in different studies [24]. While the protective effects of BS and subsequent weight reduction against GDM are apparent, specific bariatric procedures, like rugby, may affect glucose metabolism and have implications for pregnancy success and GDM detection. Obstetricians should exercise vigilance in monitoring such cases [24].

Challenges in Diagnosis Post-BS

Research on pregnant women undergoing gastric bypass surgery reveals that while fasting glucose levels improve, postprandial glucose dynamics exhibit distinct patterns. In more than 50% of pregnant individuals, there is an observed increase in blood glucose levels at the 60-minute mark, which is subsequently followed by a drop in blood sugar levels, resulting in hypoglycemia around the 120-minute mark [20]. Obstetricians ought to explore alternative diagnostic methods, like regular capillary blood glucose testing or continuous subcutaneous glucose monitoring, despite the absence of established guidelines [21]. Understanding the signs of dumping syndrome, which manifests within 15 minutes to an hour after a high-sugar meal, is crucial for obstetricians [25,26].

Late dumping syndrome, which occurs two to three hours after a meal, results in symptoms such as sweating, trembling, altered consciousness, palpitations, and syncope [27,28]. Although strategies to manage these symptoms include dietary changes and increasing food viscosity with substances like pectin or guar gum, these options often prove unpalatable and underutilized. While somatostatin analogs and acarbose have been explored in non-pregnant individuals, their application during pregnancy remains limited. Notably, diazoxide, which reduces insulin release and shows promise in alleviating late dumping syndrome, is not considered safe during pregnancy. Obstetricians encountering symptoms suggestive of dumping syndrome should collaborate with bariatric specialists for optimal patient management [28].

Hypertension and preeclampsia in the context of pregnancy

Pregnancy can bring on hypertensive illnesses such as preeclampsia, pregnancy-induced hypertension (PIH), and pre-existing chronic hypertension. Whenever hypertension is seen, defined as a systolic blood pressure greater than 140 mmHg or a diastolic blood pressure greater than 90 mmHg, preeclampsia is often diagnosed after the 20th week of pregnancy. Proteinuria, alongside maternal organ dysfunction, including renal insufficiency, hepatic involvement, or cognitive disorders, or as a consequence of blood abnormalities stemming from fetal growth retardation, must also be concurrently evident. Approximately 10% of all pregnancies are impacted by all of these disorders, which also account for 14% of all maternal fatalities globally. A significant development is the rise in preeclampsia-related hospital deliveries, which has increased by 21% in the United States. Disturbingly, obese mothers are three times more likely to develop preeclampsia compared to mothers of average weight [12,13,29]. One intriguing hypothesis is that women who conceive after undergoing BS may experience a reduced incidence of hypertension-related issues, given that BS stands as one of the most effective obesity treatments. Indeed, existing evidence supports this hypothesis [15]. A comparative study of women who gave birth before and after BS highlights a noteworthy contrast. Over 15% of women who conceived before BS experienced preeclampsia, while only 3% of those who developed after the surgery were affected. Furthermore, the post-surgery group exhibited a 75% lower risk of being diagnosed with hypertensive diseases thanks to significantly lower rates of PIH (2.5% compared to 13.0%). Numerous reviews and meta-analyses have reached similar conclusions, reporting an overall odds ratio of 0.42. When conception occurred within the first two years following surgery, it plummeted to 0.14 for hypertension problems during pregnancies following BS [7,30].

While the available data suggests a reduced risk of hypertensive disorders in pregnant individuals following BS compared to obese non-surgical controls, it is essential to note that the risk remains higher than that of normal-weight women who have not undergone such surgery. It's worth mentioning that only studies focusing on LAGB found substantial evidence that preeclampsia rates were lower in women post-BS, with no discernible difference regarding PIH [10]. Further research is warranted, particularly regarding the influence of different surgical procedures and the interval between surgery and conception. Nevertheless, the existing data underscores the substantially decreased risk of hypertensive issues for pregnant individuals who have undergone BS [12,31].

Surgical complications

Women who have undergone BS may face an increased risk of internal herniation during pregnancy. This susceptibility arises due to the enlargement of the uterus, which elevates the colon and raises intra-abdominal pressure. In cases of severe abdominal discomfort during pregnancy, it is imperative to consider immediate surgical intervention, even if the incubation must be continued [32]. After an RYGB, internal hernias are a significant concern because they can develop in as many as 10% of cases. The transverse mesocolon defect, Petersen's gap, and the mesenteric defect beneath the jejuno-jejunal anastomosis are the most frequently occurring sites for internal hernia development [33,34].

**Figure 2:**
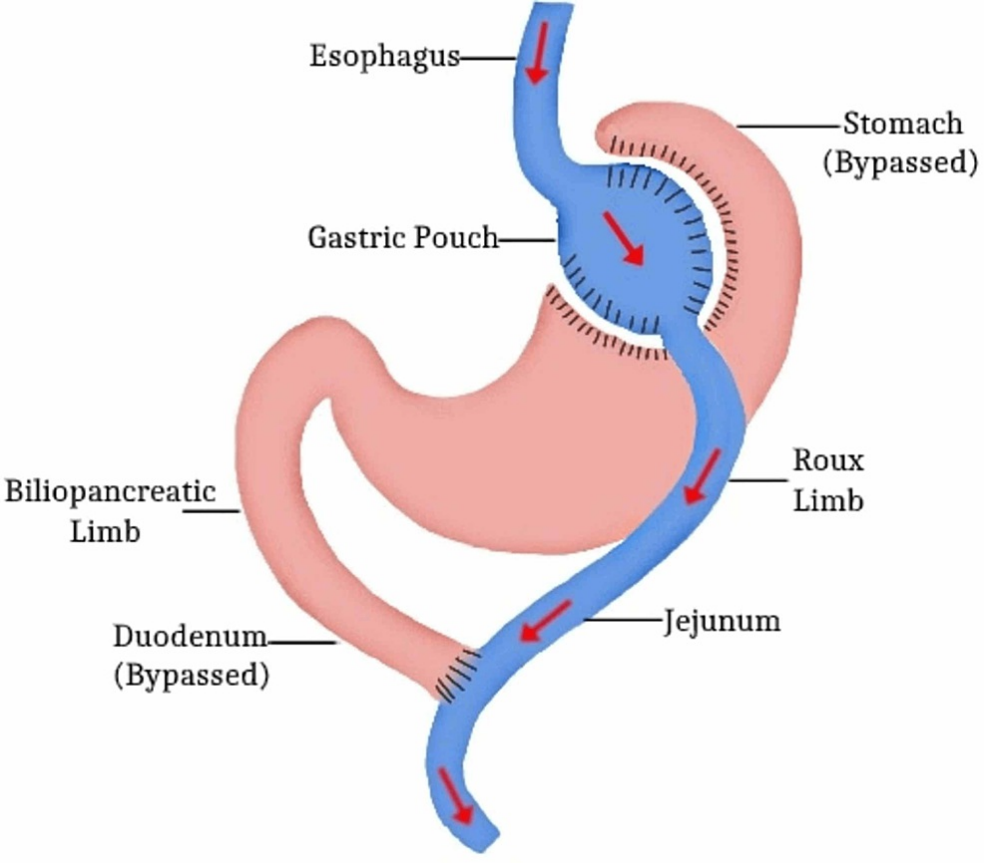
Roux-en-Y Gastric Bypass Credit: Image created by the author

A distinct form of internal hernia, Petersen's hernia, involves the small intestine protruding into the gap between the edge of the Roux limb and the lower surface of the transverse mesocolon. This retro anastomotic hernia can swiftly lead to severe bowel obstruction and necrosis. In the event of a suspected internal hernia, a crucial emergency operation becomes necessary, and fasting is mandated while closely observing the patient by the mentioned guidelines [33]. A subacute procedure should be contemplated when abdominal pain reoccurs after meals. However, should the discomfort persist despite fasting, an urgent scenario involving detorsion or colon resection becomes imperative and should be promptly executed. This timely response is vital in minimizing the risks associated with severe maternal and fetal complications and preventing intestinal necrosis [34]. RYGB is shown in Figure 2.

Fetal abnormalities in the context of maternal obesity

The impact of maternal obesity during pregnancy goes beyond the well-documented health concerns for the mother. Recent studies have highlighted potential risks to the developing fetus, including a higher likelihood of experiencing specific congenital malformations. These anomalies include orofacial clefts, genetic heart issues, and neurological disorders. A comprehensive systematic review and meta-analysis have provided valuable insights by investigating the risk of congenital abnormalities in newborns born to pregnant women who are obese. The study found that specific congenital anomalies, such as anorectal atresia (with an odds ratio of 1.48 and a confidence interval of 1.12-1.97), cardiovascular defects (with an odds ratio of 1.30 and a confidence interval of 1.12-1.51), and spina bifida are more prevalent in neonates of obese mothers when compared to their lean counterparts.

These findings are consistent with recent research, highlighting the association between maternal obesity and an increased risk of congenital abnormalities. However, despite the compelling data, the mechanisms underlying the link between obesity and fetal anomalies remain elusive. The complexity of this issue is compounded by the challenges associated with prenatal diagnosis during the early stages of pregnancy, particularly in cases involving obese women. Understanding the intricate relationship between maternal obesity and fetal abnormalities necessitates further investigation. To address this crucial gap in knowledge, continued research efforts are imperative. By unraveling the complex web of factors at play, we can hope to develop strategies that mitigate the risks associated with maternal obesity and ultimately improve the well-being of both mothers and their unborn children.

Newborn and obstetric issues

It is well-recognized that maternal obesity can result in LGA kids, which run a high risk of birth problems such as shoulder dystocia, as well as long-term health effects like childhood obesity, diabetes, and heart disease [25]. In contrast to exclusively restrictive treatments, some studies have discovered a higher incidence of SGA children delivered to moms who underwent malabsorptive or combined bariatric procedures [24]. SGA fetuses appear to be associated with reduced maternal glucose levels during OGTTs or glucose challenges. However, the exact underlying mechanism of this phenomenon is still unclear. Our research team most recently discovered a link between reduced newborn weight, a low glucose level, and higher insulin release following an OGTT in children of women who had rugby. There is a substantial inverse link between maternal weight loss throughout pregnancy and surgery and newborn weight and length (the lower the birth weight and length, the higher the weight loss). Infants from RYGB moms also had low levels of the hormone leptin and insulin-like growth factor-1 in their cord blood, which may have indicated that their anabolism was lower [26].

**Figure 3:**
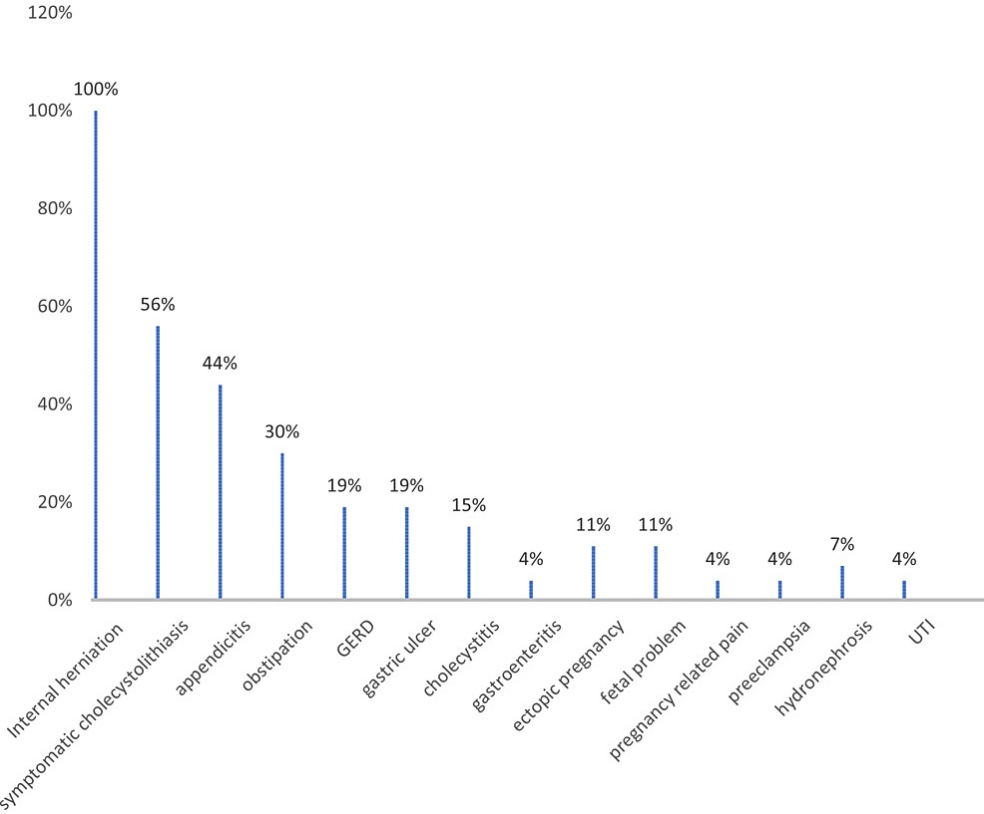
Differential diagnoses in pregnant women with acute abdominal pain after bariatric surgery GERD: Gastro-esophageal reflux disease; UTI: Urinary tract infection Credit: Image created by the author

The long-term effects of low birth weight continue to affect children even into adulthood negatively. It is thought that having an SGA condition at birth increases the risk of developing insulin resistance, metabolic syndrome, type 2 diabetes, and cardiovascular diseases. This is probably because of prenatal programming brought on by alterations in the intrauterine environment in impoverished mothers. Therefore, it can be suggested that young women who want to become mothers favor restricted BS methods over malabsorptive ones to prevent those issues [18]. However, two retrospective investigations comparing fetal birth weight following restrictive and malabsorptive surgeries in Israel and France revealed no statistically significant difference in SGA rates between the two groups [17,18]. Graph of differential diagnoses of gynecological-related problems, gastrointestinal, and other issues are shown in Figure 3.

Breastfeeding: the nutritional elixir for infants

Human breast milk is a remarkable elixir, composed of 87% water, 3.8% fat, 1.0% protein, and 7% lactose, and it serves as a bountiful source of essential nutrients. Beyond these macronutrients, breast milk offers many vitamins, minerals, digestive enzymes, hormones, and bioactive substances. Among these bioactive compounds, human milk oligosaccharides have been discovered to harbor anti-bacterial properties, providing vital support to an infant's developing digestive system. Except for vitamins D and K, human breast milk provides an adequate supply of all essential vitamins, ensuring a micronutrient-rich diet for the newborn [35,36]. However, the absence of these two vitamins poses a risk of vitamin deficiency for the infant. In cases where mothers have undergone gastric bypass surgery, breastfed infants may be at risk of vitamin B12 deficiency, which can lead to adverse consequences such as polycythemia or megaloblastic anemia. Furthermore, there have been instances where breastfeeding mothers post-gastric bypass surgery have been found to produce milk with reduced nutritional density, particularly in terms of milk fats, potentially causing growth delays in exclusively breastfed infants [37].

Nevertheless, it is imperative to acknowledge the numerous advantages of breastfeeding. Extensive research has established that breastfeeding offers protection against a range of health conditions, including atopic, cardiovascular, and infectious disorders. This includes a lower incidence of respiratory infections, asthma, leukemia, and sudden infant death syndrome. Additionally, breastfeeding has been linked to favorable effects on brain and neural development, with potential associations with higher intelligence quotient levels. Studies have suggested that exclusive breastfeeding for a duration exceeding six months may reduce the risk of future obesity [16]. Given this wealth of benefits, it is advisable to encourage bariatric patients to breastfeed their infants, as there is limited evidence to support significant nutritional deficits in breast milk following BS [38-40]. In most cases, the numerous advantages inherent in human breast milk far outweigh any potential shortcomings stemming from BS. Vigilance and support in this regard can safeguard the health and development of breastfeeding infants [15,16,18,23].

Results and discussion

BS has witnessed substantial advancements in recent years, offering effective solutions for individuals grappling with severe obesity and its associated comorbidities. The review delves into various aspects of bariatric procedures, including the recommended criteria for considering BS, surgical techniques, and the role of minimally invasive endoscopic methods. Furthermore, it explores the obstacles and advantages of BS before conception, touching upon issues like anemia, vitamin deficiencies, glucose metabolism, and gestational diabetes. The review also addresses surgical complications that may arise in pregnant women who have undergone BS [18,19,35].

BS has emerged as a paramount option for individuals without success with other weight-loss methods. The potential benefits of BS extend beyond weight loss, encompassing the amelioration of comorbidities like type 2 diabetes and hypertension and improvements in heart function. The international consensus supports the consideration of BS when a patient's BMI exceeds 40 kg/m^2^, falls within the range of 35 to 40 kg/m^2^ with severe comorbidities, or even when the BMI lies between 30 and 35 kg/m^2^. Regarding pregnancy after BS, the review highlights the significance of addressing complications and nutritional deficiencies that may affect both mothers and offspring [16]. Anemia, vitamin B12, and folic acid deficiencies pose concerns, and the altered maternal glucose metabolism can influence gestational diabetes. BS may impact fetal outcomes, with a potential risk of SGA children, internal hernias, and other surgical complications [3,8,19,27].

Furthermore, maternal obesity is discussed, emphasizing the increased risk of hypertensive disorders and their implications for pregnancy. The review acknowledges the potential reduction in the risk of hypertensive issues in pregnant individuals following BS. However, further research is needed to explore the impact of different surgical techniques and the timing of conception. The comprehensive examination of these critical aspects of BS, pregnancy, and maternal obesity underscores the multifaceted nature of these interrelated factors [38,39,41-43]. While BS has proven effective in addressing obesity and comorbidities, it introduces complex challenges and considerations for women of childbearing age. The review provides a valuable resource for healthcare practitioners and researchers, emphasizing the importance of personalized care and vigilant monitoring in pregnant individuals who have undergone BS. Addressing these complexities is paramount in ensuring the well-being of both, mothers and their offspring, and further research is warranted to refine our understanding of these intricate relationships [44-48]. A comparative analysis of all the studies included in the review is described in Table 1.

**Table 1:** Comparative analysis of all the studies included in the review IFSO-EC: International Federation for the Surgery of Obesity and Metabolic Disorders - European Chapter; EASO: European Association for the Study of Obesity; EBMT: Endoscopic Bariatric and Metabolic Therapies; CD: Cesarean delivery; RCT: Randomized controlled trial; T2DM: Type 2 diabetes mellitus, GDM: Gestational diabetes mellitus, IDA: Iron deficiency anemia, PCOS: Polycystic ovary syndrome; MOSH: Male Obesity Associated Secondary Hypogonadism; HOMA-IR: Homeostatic Model Assessment of Insulin Resistance; SGA: Small for gestational age; LBA: Large for gestational age; BMI: Body mass index

Author	Journal and year of publication	Interpretation
Arterburn et al. [1]	JAMA, 2020	Modern bariatric procedures are safe and effective, particularly for patients with severe obesity and type 2 diabetes. It is crucial to involve these patients in shared decision-making discussions regarding the choice between surgery and traditional medical and lifestyle treatments. Ultimately, the decision should prioritize the patient's well-informed preferences.
Damti et al. [2]	Arch Gynecol Obstet, 2019	Children of bariatric surgery patients and obese moms are more likely to experience long-term pediatric endocrine morbidity.
Stroup et al. [3]	JAMA, 2000	The checklist covers the background, search strategy, methodology, findings, discussion, and conclusion and specifies the requirements for publishing observational epidemiology meta-analyses. The usefulness of meta-analyses is increased for writers, editors, reviewers, readers, and decision-makers through its implementation. It also looks at research directions and suggests an evaluation methodology.
Jensen et al. [4]	J Am Coll Cardiol, 2014	Bariatric surgery offers safe and effective weight loss solutions, particularly for individuals with severe obesity and type 2 diabetes. Shared decision-making is crucial, involving a discussion of the surgery's pros and cons versus conventional medical and lifestyle approaches. Ultimately, the patient's informed preferences should primarily guide the choice for surgery.
Fried et al. [5]	Obes Surg, 2014	In 2012, experts in metabolic and bariatric surgery, with input from IFSO-EC and EASO, convened to update the 2008 European guidelines on severe obesity surgery. Their focus was on recent developments, particularly regarding diabetes, and they aimed to integrate the latest evidence for current, expert-driven management of obesity and related issues.
Catalano et al. [6]	BMJ, 2017	Maternal obesity in pregnancy leads to various short and long-term issues for both mother and child, including infertility, gestational diabetes, and childhood obesity.
Stothard et al. [7]	JAMA, 2009	A number of anatomical malformations are more likely to occur in mothers who are obese; however, the absolute increase is probably not very great. To determine whether maternal overweight is also involved, more research is required.
Catalano et al. [8]	Am J Obstet Gynecol, 2011	Pedersen's hypothesis, over 50 years old, linked fetal overgrowth to increased glucose transfer in diabetes. Understanding these aids in addressing obesity-related perinatal metabolic issues.
Schauer et al. [9]	Cleve Clin J Med, 2017	Metabolic surgery treats type 2 diabetes and metabolic issues effectively, surpassing lifestyle and medical methods. It's an option for lower body mass index patients.
Karason et al. [10]	Obes Res, 1998	Weight loss in obesity improves left ventricular function, with no apparent link to valvular heart disease.
Elder et al. [11]	Gastroenterology, 2007	Obesity's rise calls for bariatric surgery's consideration. It proves effective, but access inequalities persist. Enhanced presurgical assessment and research are vital.
Sullivan et al. [12]	Gastroenterology, 2017	EBMT offers innovative approaches to obesity treatment. They include gastric and small bowel methods with diverse mechanisms. They were discussing approved therapies, safety, and efficacy.
Barker et al. [13]	Obes Rev, 2007	Childhood obesity and later disease are linked to early growth and birth size. Preventing childhood obesity requires improving infant nutrition. Monitoring trends is crucial.
Chevrot et al. [14]	Am J Obstet Gynecol, 2016	Malabsorptive bariatric surgery was associated with an increased risk of fetal growth restriction.
Izquierdo et al. [15]	Front Endocrinol (Lausanne), 2019	The obesity-related epigenome can be partially reversed by bariatric surgery. Finding putative epigenetic biomarkers that predict bariatric surgery outcomes might lead to more individualized treatments for extreme obesity.
Grange et al. [16]	Pediatr Hematol Oncol, 1994	Infants of mothers with gastric bypass surgery can develop vitamin B12 deficiency. Supplementation for mothers during and after pregnancy is essential to prevent this.
Adam et al. [17]	BMJ, 2017	The study examined fetal outcomes in gestational diabetic women. Early glucose tolerance screening can help prevent complications in those with risk factors.
Simeoni et al. [18]	Semin Fetal Neonatal Med, 2009	Diabetes during pregnancy impacts offspring, leading to hypertension, T2DM, and obesity. This review explores the mechanisms and lifelong consequences.
Bonouvrie et al. [19]	Bariatr Surg Pract Patient Care, 2022	Inconsistent practice prevails for pregnancy in bariatric surgery. Acute complications pose challenges, and referrals to specialized centers are lacking. Improved information and guidance are essential.
Isaacs et al. [20]	Pediatr Res, 2010	Breast milk's influence on brain growth and cognitive development is supported, particularly affecting the white matter in boys, with significant gender-related effects.
Sweet et al. [21]	Midwifery, 2022	Informing healthcare providers and women about post-bariatric surgery pregnancy and lactation is crucial. Tailored care and more research are needed.
Faria et al. [22]	Obes Surg, 2011	Bariatric surgery patients need proper protein intake for satiety, weight loss, and health. The optimal protein quantity and quality require further research.
Fetita et al. [23]	J Clin Endocrinol Metab, 2006	Maternal diabetes exposure in utero may contribute to the diabetes epidemic. Targeting high-risk populations and improving glycemic control during pregnancy is essential.
Lekva et al. [24]	Curr Diab Rep, 2016	GDM prevalence is rising, with risks for mothers and children. Inflammation's role in GDM pathogenesis is explored in this monograph.
Mcardle et al. [25]	Proc Nutr Soc, 2014	This review underscores placental importance in fetal iron metabolism. Iron deficiency during pregnancy affects both mother and baby, warranting consideration of iron prophylaxis.
Galazis et al. [26]	Eur J Obstet Gynecol Reprod Biol, 2014	Bariatric surgery improves pregnancy outcomes, with laparoscopic adjustable gastric banding showing no increased risk of small neonates. Obese women should consider these findings.
Milman et al. [27]	Ann Hematol, 2008	IDA is common in pregnancy, affecting 14-52% of women without iron supplements. Oral or intravenous iron treatment is effective.
Escobar-Morreale et al. [28]	Hum Reprod Update, 2017	Severely obese patients with gonadal dysfunction, often linked to PCOS or MOSH, should be offered bariatric surgery due to the positive outcomes observed.
Nestler et al. [29]	N Engl J Med, 2008	The increasing prevalence of GDM carries risks during pregnancy and in the long term. Inflammation's role in GDM pathogenesis is explored in this monograph.
Gilead et al. [30]	J Matern Fetal Neonatal Med, 2012	Isolated obesity isn't a risk for perinatal issues but independently raises the risk of CD.
Johansson et al. [31]	N Engl J Med, 2015	Out of 3,470 initial search results, 33 studies met the criteria, assessing perinatal outcomes in pregnancies after bariatric surgery and comparing them with controls.
Smid et al. [32]	Obes Surg, 2017	Many obstetricians lack awareness of appropriate nutrition and monitoring practices for pregnant women post-bariatric surgery. Education is crucial to address this gap.
Hill et al. [33]	Ann N Y Acad Sci, 2018	Bariatric endoscopy offers minimally invasive treatment for obesity and its comorbidities, bridging the gap between counseling, medication, and surgery. Multiple devices are approved and in development.
Marchi et al. [34]	Obes Rev, 2015	Maternal obesity increases risks during pregnancy, including gestational diabetes, pre-eclampsia, preterm birth, and breastfeeding challenges, necessitating proactive interventions.
Russo et al. [35]	Obes Surg, 2007	Severe obesity surgery reduces ventricular repolarization variability, potentially lowering the risk of life-threatening arrhythmias in morbidly obese individuals.
Galazis et al. [36]	Am J Obstet Gynecol, 2016	Even though it is a risk factor for cesarean birth on its own, prior bariatric surgery is not linked to poor perinatal outcomes.
Amsalem et al. [37]	Surg Obes Relat Dis, 2014	Restrictive bariatric surgery substantially reduces pregnancy complications like hypertension and gestational diabetes, with lasting benefits during subsequent pregnancies.
Aricha-Tamir et al. [38]	Surg Obes Relat Dis, 2012	Bariatric surgery significantly reduces pregnancy complications, including hypertension and diabetes mellitus.
Dalfrà et al. [39]	J Matern Fetal Neonatal Med, 2012	Maternal obesity harms offspring. Bariatric surgery helps morbidly obese pregnant women, but risks vary by procedure. Long-term studies show improved child obesity rates - specialized care is needed for pregnancies post-surgery.
Lapolla et al. [40]	J Endocrinol Invest, 2018	The gestational pathway shows success with increased utilization, particularly among immigrant women. The pre-gestational path is rarely used. Collaboration and local resources are crucial, but preventing obesity in fertile women remains a challenge.
Spencer et al. [41]	JBI Database System Rev Implement Rep, 2015	This systematic review assesses diet-based weight management interventions' effectiveness in pregnant and postpartum women, examining various intervention components and their impact on maternal weight outcomes. No existing reviews cover this topic.
Qu et al. [42]	Food Sci Nutr, 2022	A meta-analysis of four RCTs (198 participants) found that magnesium supplementation in gestational diabetes mellitus patients significantly improved glycemic control and reduced insulin levels, positively impacting insulin sensitivity and oxidative stress.
Zhang et al. [43]	J Diabetes Res, 2019	Probiotic supplementation benefits pregnant women with GDM by reducing newborn hyperbilirubinemia and improving various health aspects, but more diverse studies are needed for broader applicability.
Łagowska et al. [44]	Sci Rep, 2020	This meta-analysis assesses probiotic and synbiotic effects on glucose metabolism in pregnant women, primarily those with GDM, demonstrating benefits in lowering glucose, insulin, and HOMA-IR levels. Further research with larger cohorts is needed for better estimation.
Bretón et al. [45]	J Clin Med, 2023	Bariatric surgery is increasingly common in women of childbearing age. This review analyzes how various procedures affect mineral and micronutrient status during pregnancy and maternal-fetal health.
Yu et al. [46]	Plos One, 2013	Pre-pregnancy underweight raises SGA and LBW risk, while overweight/obesity raises LGA, HBW, macrosomia, and offspring overweight/obesity risk. Future studies should explore potential modifiers.
Frederick et al. [47]	Matern Child Health J, 2008	Findings emphasize the importance of considering pre-pregnancy weight and gestational weight gain to manage LBW and macrosomia risks in lean and obese women.
Gudipally et al. [48]	AJOG Glob Rep, 2022	Elevated pre-pregnancy BMI increases the risk of adverse outcomes, notably cesarean delivery. While our study focuses on Indian regions, further research and intervention programs are needed for overweight and obese women.

## Conclusions

There are many concerns for both the mother and the fetus when there is a history of BS. Preconception counseling is recommended for women who desire to get pregnant in order to educate them about the dangers of having a baby after BS, including internal hernias, nutritional deficiencies, and SGA babies. It is essential to undertake routine blood tests and ultrasounds of the developing fetus (growing-curve, umbilical Doppler, amniotic fluid index). Additionally, because to the significant risk of hypoglycemia, the OGTT should not be used as a regular test for the screening of gestational diabetes. Pregnant women should ideally get care from a specialist facility with a multidisciplinary staff with knowledge of managing pregnancies following BS.

Due to the increased likelihood of internal hernia, any acute upper abdominal discomfort must be addressed carefully. Due to the novelty of pregnancy after BS, a worldwide therapeutic consensus is currently lacking; as a result, precise guidelines for delivery methods or nursing are not yet available. Obstetricians may have more difficulties in the coming years as a result of the rise in the number of pregnant BS patients and potential complications.

## References

[1] Arterburn DE, Telem DA, Kushner RF, Courcoulas AP (2020). Benefits and risks of bariatric surgery in adults: a review. JAMA.

[2] Damti P, Friger M, Landau D, Sergienko R, Sheiner E (2019). Offspring of women following bariatric surgery and those of patients with obesity are at an increased risk for long-term pediatric endocrine morbidity. Arch Gynecol Obstet.

[3] Stroup DF, Berlin JA, Morton SC (2000). Meta-analysis of observational studies in epidemiology: a proposal for reporting. Meta-analysis of Observational Studies in Epidemiology (MOOSE) group. JAMA.

[4] Jensen MD, Ryan DH, Apovian CM (2014). 2013 AHA/ACC/Tos guideline for the management of overweight and obesity in adults: a report of the American College of Cardiology/American Heart Association Task Force on practice guidelines and the Obesity Society. J Am Coll Cardiol.

[5] Fried M, Yumuk V, Oppert JM (2014). Interdisciplinary European guidelines on metabolic and bariatric surgery. Obes Surg.

[6] Catalano PM, Shankar K (2017). Obesity and pregnancy: mechanisms of short term and long term adverse consequences for mother and child. BMJ.

[7] Stothard KJ, Tennant PW, Bell R, Rankin J (2009). Maternal overweight and obesity and the risk of congenital anomalies: a systematic review and meta-analysis. JAMA.

[8] Catalano PM, Hauguel-De Mouzon S (2011). Is it time to revisit the Pedersen hypothesis in the face of the obesity epidemic?. Am J Obstet Gynecol.

[9] Schauer PR, Nor Hanipah Z, Rubino F (2017). Metabolic surgery for treating type 2 diabetes mellitus: now supported by the world's leading diabetes organizations. Cleve Clin J Med.

[10] Karason K, Wallentin I, Larsson B, Sjöström L (1998). Effects of obesity and weight loss on cardiac function and valvular performance. Obes Res.

[11] Elder KA, Wolfe BM (2007). Bariatric surgery: a review of procedures and outcomes. Gastroenterology.

[12] Sullivan S, Edmundowicz SA, Thompson CC (2017). Endoscopic bariatric and metabolic therapies: new and emerging technologies. Gastroenterology.

[13] Barker DJ (2007). Obesity and early life. Obes Rev.

[14] Chevrot A, Kayem G, Coupaye M, Lesage N, Msika S, Mandelbrot L (2016). Impact of bariatric surgery on fetal growth restriction: experience of a perinatal and bariatric surgery center. Am J Obstet Gynecol.

[15] Izquierdo AG, Crujeiras AB (2019). Obesity-related epigenetic changes after bariatric surgery. Front Endocrinol (Lausanne).

[16] Grange DK, Finlay JL (1994). Nutritional vitamin B12 deficiency in a breastfed infant following maternal gastric bypass. Pediatr Hematol Oncol.

[17] Adam S, Ammori B, Soran H, Syed AA (2017). Pregnancy after bariatric surgery: screening for gestational diabetes. BMJ.

[18] Simeoni U, Barker DJ (2009). Offspring of diabetic pregnancy: long-term outcomes. Semin Fetal Neonatal Med.

[19] Bonouvrie DS, Taverne SB, Janssen L, Luijten AA, van Dielen FM, Leclercq WK (2022). Pregnancy and bariatric surgery: significant variation in bariatric surgeons’ practices and preferences: a national survey. Bariatr Surg Pract Patient Care.

[20] Isaacs EB, Fischl BR, Quinn BT, Chong WK, Gadian DG, Lucas A (2010). Impact of breast milk on IQ, brain size and white matter development. Pediatr Res.

[21] Sweet L, Vasilevski V (2022). Women's experiences of pregnancy and lactation after bariatric surgery: a scoping review. Midwifery.

[22] Faria SL, Faria OP, Buffington C, de Almeida Cardeal M, Ito MK (2011). Dietary protein intake and bariatric surgery patients: a review. Obes Surg.

[23] Fetita LS, Sobngwi E, Serradas P, Calvo F, Gautier JF (2006). Consequences of fetal exposure to maternal diabetes in offspring. J Clin Endocrinol Metab.

[24] Lekva T, Norwitz ER, Aukrust P, Ueland T (2016). Impact of systemic inflammation on the progression of gestational diabetes mellitus. Curr Diab Rep.

[25] McArdle HJ, Gambling L, Kennedy C (2014). Iron deficiency during pregnancy: the consequences for placental function and fetal outcome. Proc Nutr Soc.

[26] Galazis N, Docheva N, Simillis C, Nicolaides KH (2014). Maternal and neonatal outcomes in women undergoing bariatric surgery: a systematic review and meta-analysis. Eur J Obstet Gynecol Reprod Biol.

[27] Milman N (2008). Prepartum anaemia: prevention and treatment. Ann Hematol.

[28] Escobar-Morreale HF, Santacruz E, Luque-Ramírez M, Botella Carretero JI (2017). Prevalence of 'obesity-associated gonadal dysfunction' in severely obese men and women and its resolution after bariatric surgery: a systematic review and meta-analysis. Hum Reprod Update.

[29] Nestler JE (2008). Metformin for the treatment of the polycystic ovary syndrome. N Engl J Med.

[30] Gilead R, Yaniv Salem S, Sergienko R, Sheiner E (2012). Maternal "isolated" obesity and obstetric complications. J Matern Fetal Neonatal Med.

[31] Johansson K, Cnattingius S, Näslund I (2015). Outcomes of pregnancy after bariatric surgery. N Engl J Med.

[32] Smid MC, Dotters-Katz SK, Mcelwain CA, Volckmann ET, Schulkin J, Stuebe AM (2017). Pregnancy after bariatric surgery: national survey of obstetrician’s comfort, knowledge, and practice patterns. Obes Surg.

[33] Hill C, Khashab MA, Kalloo AN, Kumbhari V (2018). Endoluminal weight loss and metabolic therapies: current and future techniques. Ann N Y Acad Sci.

[34] Marchi J, Berg M, Dencker A, Olander EK, Begley C (2015). Risks associated with obesity in pregnancy, for the mother and baby: a systematic review of reviews. Obes Rev.

[35] Russo V, Ammendola E, De Crescenzo I (2007). Effect of weight loss following bariatric surgery on myocardial dispersion of repolarization in morbidly obese patients. Obes Surg.

[36] Galazis N, Sein E (2016). Restrictive bariatric procedures improve pregnancy outcomes compared with malabsorptive procedures. Am J Obstet Gynecol.

[37] Amsalem D, Aricha-Tamir B, Levi I, Shai D, Sheiner E (2014). Obstetric outcomes after restrictive bariatric surgery: what happens after 2 consecutive pregnancies?. Surg Obes Relat Dis.

[38] Aricha-Tamir B, Weintraub AY, Levi I, Sheiner E (2012). Downsizing pregnancy complications: a study of paired pregnancy outcomes before and after bariatric surgery. Surg Obes Relat Dis.

[39] Dalfrà MG, Busetto L, Chilelli NC, Lapolla A (2012). Pregnancy and foetal outcome after bariatric surgery: a review of recent studies. J Matern Fetal Neonatal Med.

[40] Lapolla A, Scibetta D, Gallina P (2018). Innovative clinical pathways for obese pregnant women: design and feasibility of the Padua project (north-eastern Italy). J Endocrinol Invest.

[41] Spencer L, Rollo M, Hauck Y (2015). The effect of weight management interventions that include a diet component on weight-related outcomes in pregnant and postpartum women: a systematic review protocol. JBI Database System Rev Implement Rep.

[42] Qu Q, Rong R, Yu J (2022). Effect of magnesium supplementation on pregnancy outcome in gestational diabetes mellitus patients: a meta-analysis of randomized controlled trials. Food Sci Nutr.

[43] Zhang J, Ma S, Wu S, Guo C, Long S, Tan H (2019). Effects of probiotic supplement in pregnant women with gestational diabetes mellitus: a systematic review and meta-analysis of randomized controlled trials. J Diabetes Res.

[44] Łagowska K, Malinowska AM, Zawieja B, Zawieja E (2020). Improvement of glucose metabolism in pregnant women through probiotic supplementation depends on gestational diabetes status: meta-analysis. Sci Rep.

[45] Bretón I, Ballesteros-Pomar MD, Calle-Pascual A, Alvarez-Sala LA, Rubio-Herrera MA (2023). Micronutrients in pregnancy after bariatric surgery: a narrative review. J Clin Med.

[46] Yu Z, Han S, Zhu J, Sun X, Ji C, Guo X (2013). Pre-pregnancy body mass index in relation to infant birth weight and offspring overweight/obesity: a systematic review and meta-analysis. PLoS One.

[47] Frederick IO, Williams MA, Sales AE, Martin DP, Killien M (2008). Pre-pregnancy body mass index, gestational weight gain, and other maternal characteristics in relation to infant birth weight. Matern Child Health J.

[48] Gudipally M, Farooq F, Basany K (2023). Impact of prepregnancy body mass index on adverse pregnancy outcomes: analysis from the Longitudinal Indian Family hEalth cohort study. AJOG Glob Rep.

